# Editorial: Fertility in Klinefelter Syndrome: The Need for a Multidisciplinary Approach

**DOI:** 10.3389/frph.2021.792479

**Published:** 2021-11-24

**Authors:** Tet Yap

**Affiliations:** Guy's and St. Thomas' Foundation Trust, London, United Kingdom

**Keywords:** Klinefelter Syndrome, fertility, gynaecomastia, testis (MeSH), sperm, surgical sperm retrieval, fertility preservation, XXY karyotype

Klinefelter Syndrome (KS) is the commonest disorder that affects the sex chromosomes (1), occurring in one in 660 newborn males (2). The non-mosaic 47XXY chromosomal arrangement is present in 80% of KS patients (1). It can present in the mosaic form (47,XXY/46,XY) as well, often with less phenotypic severity (1). Men are often diagnosed when they present with primary infertility. KS patients represent 3% of infertile males and 10% of azoospermic men (2–4). Sperm in the ejaculate has been identified in up to 70% semen samples from adolescents with KS aged from 12 to 20 years (5, 6). However, in late adolescence, testosterone levels fall and leading to an absence in secondary sexual characteristics, small testes and gynaecomastia with the progressive fibrosis and hyalinisation of the seminiferous tubules and spermatogonial stem cell decline into adulthood, leading to azoospermia (7).

KS is a multisystem disease that does not just impact sperm production. Many issues surrounding hypogonadism, psychosexual morbidity, gynaecomastia and risk of developing further disease such as diabetes, osteoporosis, venous thromboembolism and cancer need to be discussed and require more research (8). The care of patients with KS, like other multisystem genetic disease, needs to be more multidisciplinary and integrated. This Research Topic focuses on the many often missed issues that affect patients with KS, beyond just their fertility journey.

The framework for the development of a multidisciplinary team (MDT) based clinic is described in the paper by Espehana et al., and describes a holistic, patient-centered approach that encompasses the key specialties and support required for MDT care in these patients—endocrine, urology and fertility, genetics, psychology and psychosexual medicine with expert radiology, pharmacy and specialty nursing support. A key feature is a patient liaison from a Klinefelter patient group, such as the KSA (Klinefelter Association) in UK. The success of the MDT approach has been quantifiable in terms of faster access to a wider set of clinicians, greater awareness of KS issues, greater patient and primary care satisfaction, and a cohesive management plan that acts as a roadmap for managing the condition (9).

Another key feature of the MDT approach is the ability to discuss abnormal findings detected during investigation of patients, such as indeterminate lesions of the testis. These are common sonographic findings in infertile men, including those affected by KS. A commentary by Potter and Prezzi, describes the characteristics of these imaged lesions often found in KS, and the challenges they present. Most have appearances of benign Leydig Cell tumors, which can be safely followed up or biopsied at the time of microsurgical sperm retrieval. Multiparametric ultrasound shows potential for improved lesion characterization and may assist in their management in future. With prompt discussion within an MDT setting, unnecessary surgery or prolonged imaging follow-up could be avoided in the presence of lesions with benign sonographic features; with prompt organ sparing surgery directed to lesions with malignant features.

Fertility topics are explored in two papers, Kailash et al.'s review of surgical sperm retrieval (SSR) in KS, and Pook et al.'s analysis of fertility preservation in the pediatric KS setting. Both papers highlight the need for a holistic multidisciplinary approach in order to best optimize patients for fertility harvesting. In adults, evidence is presented that suggests that the single most significant determinant for successful SSR is the age of the patient (10–12). The endocrine influences affecting SSR outcomes are also explored, with a review of evidence linking response to hormone stimulation with higher success rates (4, 13). Several studies have reported live births of healthy offspring using this retrieved sperm from KS men, which is promising (14). A protocol for hormone stimulation based on published and institutional experience is suggested, along with a call for more studies to standardize protocols and further confirm the efficacy of these treatments. The need for adequate counseling and the role of endocrine manipulation further supports the need for multidisciplinary care. This is just as evident in Pook et al.'s review. The current focus of pediatric KS treatment involves targeting endocrine deficits to improve growth, muscle mass, and prevent delayed puberty. As discussed in the preceding paper on SSR in adult KS, patients with KS and NOA demonstrate a time-sensitive pattern of germ cell depletion, suggesting younger age is a potential positive predictor of success in sperm retrieval (15). In a review of 76 studies, SRR in males with KS under the age of 16 years was low at 0–20% but increased to 40–70% in those aged between 16 and 30 years (16) Bryson et al. (12) conducted a large retrospective series which found that SSR in males with KS under 30 years was significantly higher than those older than 30 years (81 vs. 33%, *p* < 0.01). Although these results appeared to be encouraging, limited sample sizes and the wide range of SSR rates is likely due to variation in testosterone pre-treatment, mosaicism/non-mosaicism, and level of testicular dysfunction. As such, no current guidelines exist on optimal timing for FP in KS, although from available evidence an age range of 16–30 is potentially optimal. The ethical considerations of fertility preservation in the pediatric age group are also discussed.

The final paper in the series looks at another often missed manifestation of KS that can have immense psychological impact. Raheem et al. reviewed the impact and management of excessive breast tissue (gynaecomastia) in KS, which affects 80% of KS patients (17). In KS, gynaecomastia is thought to be due to the increased free oestradiol and low free androgens acting on the breast tissue. An increase in the activity of aromatase enzyme which will cause an increase in the peripheral conversion of testosterone to oestradiol is also implicated. The increased risk of breast cancer (>10-fold greater in KS) (17) and its negative psychological impact (18) has rendered the assessment of gynecomastia in KS a matter of necessity rather than just aesthetics, and should be picked up when assessing fertility issues (which are the main conduit for KS patients being seen by medical professionals).

The wide range of specialist topics covered in this comprehensive review reveals the clinical issues important to KS including and beyond fertility that would ideally benefit from a multi- disciplinary team approach. This approach has now been recommended in recent guidelines (8), released after publication of this Research Topic. As a direct result of this research, and with close involvement of KS patients and KS patient bodies, the author's KS MDT service now includes a pediatric and adolescent transition KS MDT clinic, which include a psychiatrist and plastic surgeon specialized in management of gynaecomastia (19). Whilst much research is required within the individual KS specialties, close team working will ensure the best current-evidence management for KS patients and may provide a template for management of other multisystem genetic diseases.

## Author Contributions

The author confirms being the sole contributor of this work and has approved it for publication.

## Conflict of Interest

The author declares that the research was conducted in the absence of any commercial or financial relationships that could be construed as a potential conflict of interest.

## Publisher's Note

All claims expressed in this article are solely those of the authors and do not necessarily represent those of their affiliated organizations, or those of the publisher, the editors and the reviewers. Any product that may be evaluated in this article, or claim that may be made by its manufacturer, is not guaranteed or endorsed by the publisher.

## References

[1] Lanfranco F Kamischke A Zitzmann M Nieschlag PE. Klinefelter's syndrome. Lancet. (2004) 364:273–83. 10.1016/S0140-6736(04)16678-6 15262106

[2] Bojesen A Juul S Gravholt CH. Prenatal and postnatal prevalence of Klinefelter syndrome: a national registry study. J Clin Endocrinol Metabol. (2003) 88:622–6. 10.1210/jc.2002-021491 12574191

[3] Nielsen J Wohlert M. Chromosome abnormalities found amongo34910 newborn children: results from a 13-year incidence study inArhus, Denmark. Hum Genet. (1991) 87:81–3. 10.1007/BF01213097 2037286

[4] Ramasamy R Ricci JA Palermo GD Gosden LV Rosenwaks Z Schlegel PN. Successful fertility treatment for Klinefelter's syndrome. J Urol. (2009) 182:1108–13. 10.1016/j.juro.2009.05.019 19616796

[5] Mehta A Paduch DA. Klinefelter syndrome: an argument for early aggressive hormonal and fertility management. Fertil Steril. (2012) 98:274–83. 10.1016/j.fertnstert.2012.06.001 22732737

[6] Hawksworth DJ Szafran AA Jordan PW Dobs AS Herati AS. Infertility in patients with Klinefelter syndrome: optimal timing for sperm and testicular tissue cryopreservation. Rev Urol. (2018) 20:56–62. 10.3909/riu0790 30288141 PMC6168324

[7] Aksglaede L Link K Giwercman A Jørgensen N Skakkebaek NE Juul A. 47, XXY Klinefelter Syndrome: clinical characteristics and age-specific recommendations for medical management. Am J Med Genet C. (2013) 163:55–63. 10.1002/ajmg.c.31349 23345262

[8] Zitzmann M Aksglaede L Corona G Isidori AM Juul A T'Sjoen G. European academy of andrology guidelines on Klinefelter Syndrome Endorsing Organization: European Society of Endocrinology. Andrology. (2021) 9:145–67. 10.1111/andr.12909 32959490

[9] Espehana A Tomlinson C El-shirif A Prezzi D Briggs K Allchorne P. Identifying pathway delays and patient needs to optimize the management of men with adult Klinefelter Syndrome. J Urol. (2020) 203:e657. 10.1097/JU.0000000000000899.04

[10] Okada H Goda K Yamamoto Y Sofikitis N Miyagawa I Mio Y. Age as a limiting factor for successful sperm retrieval in patients with nonmosaic Klinefelter's syndrome. Fertil Steril. (2005) 84:1662–4. 10.1016/j.fertnstert.2005.05.053 16359961

[11] Rohayem J Fricke R Czeloth K Mallidis C Wistuba J Krallmann C. Age and markers of leydig cell function, but not of Sertoli cell function predict the success of sperm retrieval in adolescents and adults with Klinefelter's syndrome. Andrology. (2015) 3:868–75. 10.1111/andr.12067 26235799

[12] Bryson CF Ramasamy R Sheehan M Palermo GD Rosenwaks Z Schlegel PN. Severe testicular atrophy does not affect the success of microdissection testicular sperm extraction. J Urol. (2014) 191:175–8. 10.1016/j.juro.2013.07.065 23911635 PMC4077600

[13] Majzoub A Arafa M Al Said S Agarwal A Seif A Al Naimi A. Outcome of testicular sperm extraction in nonmosaic Klinefelter syndrome patients: what is the best approach? Andrologia. (2016) 48:171–6. 10.1111/and.12428 25929757

[14] Corona G Pizzocaro A Lanfranco F Garolla A Pelliccione F Vignozzi L. Sperm recovery and ICSI outcomes in Klinefelter syndrome: a systematic review and meta-analysis. Hum Reprod Update. (2017) 23:265–75. 10.1093/humupd/dmx008 28379559

[15] Wikström AM Raivio T Hadziselimovic F Wikström S Tuuri T Dunkel L. Klinefelter syndrome in adolescence: onset of puberty is associated with accelerated germ cell depletion. J Clin Endocrinol Metab. (2004) 89:2263–70. 10.1210/jc.2003-031725 15126551

[16] Franik S Hoeijmakers Y D'Hauwers K Braat DD Nelen WL Smeets D. Klinefelter syndrome and fertility: sperm preservation should not be offered to children with Klinefelter syndrome. Hum Reprod. (2016) 31:1952–9. 10.1093/humrep/dew179 27412247

[17] Bembo SA Carlson HE. Gynecomastia: its features, and when and how to treat it. Cleveland Clin J Med. (2004) 71:511. 10.3949/ccjm.71.6.511 15242307

[18] Nuzzi LC Cerrato FE Erikson CR Webb ML Rosen H Walsh EM. Psychosocial impact of adolescent gynecomastia: a prospective case-control study. Plastic Reconst Surg. (2013) 131:890–6. 10.1097/PRS.0b013e3182818ea8 23542261

[19] Chowdhury A Baig U Thavarajah T Arumaithurai A Absoud M Cocca A. The establishment of a multispecialty clinic for young people with Klinefelter syndrome. J Urol. (2021) 206:e823. 10.1097/JU.0000000000002068.01

